# Antimicrobial Activity and Action Approach of the Olive Oil Polyphenol Extract Against *Listeria monocytogenes*

**DOI:** 10.3389/fmicb.2019.01586

**Published:** 2019-07-23

**Authors:** Ling Guo, Qi Sun, Shaoying Gong, Xue Bi, Wen Jiang, Wei Xue, Peng Fei

**Affiliations:** ^1^ Key Laboratory of Dairy Science, Ministry of Education, College of Food Science, Northeast Agricultural University, Harbin, China; ^2^ National Agricultural Standardization Monitoring and Research Center (Heilongjiang), Harbin, China; ^3^ Metrology Institute of Measurement and Verification (Heilongjiang), Harbin, China; ^4^ College of Food and Bioengineering, Henan University of Science and Technology, Luoyang, China

**Keywords:** *Listeria monocytogenes*, olive oil polyphenol extract, antimicrobial activity, action approach, cellular morphology

## Abstract

Olive oil polyphenol extract (OOPE) has been reported to have antibacterial activity; however, its effect on *Listeria monocytogenes* is less studied so far. This study, thus, aimed to reveal its antimicrobial activity and action approach against *L. monocytogenes via* evaluating the minimum inhibitory concentration (MIC) as well as the changes of intracellular adenosine 5′-triphosphate (ATP) concentration, cell membrane potential, bacterial protein, DNA, and cell morphology. The results showed that OOPE could inhibit the growth of *L. monocytogenes* with a measured MIC of 1.25 mg/ml. *L. monocytogenes* cells treated by OOPE showed significant reduction in intracellular ATP concentrations, bacterial protein, or DNA (*p* < 0.05), in comparison with those without any treatment. In addition, OOPE was observed to depolarize strain cells and alter cell morphology, resulting in damaged cell membrane and, thereby, leakage of cell fluid. These findings demonstrated that OOPE had inhibition on *L. monocytogenes via* its action on cells, suggesting its potential as a natural preservative.

## Introduction

*Listeria monocytogenes* is a Gram-positive bacterium, and as one of the major food-borne pathogens, it is involved in some outbreaks of severe food-borne infections (Odedina et al., 2015). The survival and growth of *L. monocytogenes* are easy in the conditions of high salt concentration, low pH, and low temperature, which increase its potential as a contaminant of food products (Allen et al., 2016). Fresh vegetables and fruits, dairy products, ready-to-eat foods, and food-contact surfaces are susceptible to contamination from *L. monocytogenes* (Hamidi-Oskouei et al., 2015). Currently, a variety of technologies and safety systems are implemented to control pathogens. However, the contamination of *L. monocytogenes* is still considered as a major food safety problem (Long et al., 2011).

Compared with synthetic preservatives, natural extracts have attracted great interest due to their ability to inhibit the growth of food-borne pathogens and not trigger negative safety worries (Rendueles et al., 2011). Some plant extracts had been well documented to be used as potential natural antimicrobial agents or preservatives against *L. monocytogenes*, such as fruit and vegetable extracts from mint and pomegranate, spice extracts from cinnamon and clove, and phenolic extracts from legumes processing by-products (Aureli et al., 1992; Araya-Cloutier et al., 2018; Xylia et al., 2018). Consumers are more willing to accept natural extracts as preservatives than synthetic preservatives due to the nature and relative safety of natural products (Fei et al., 2018). Therefore, people would like to learn more information about using plant extracts as potential preservatives for controlling *L. monocytogenes*.

Polyphenols have demonstrated a series of biological effects including antibacterial, antioxidants, anti-inflammatory, antiviral, and antiallergic action (Daglia, 2012; Borges et al., 2013). Olive oil polyphenol extract (OOPE) is a natural substance obtained from olive oil and contains abundant polyphenolic compounds (Bubonja-Sonje et al., 2011). Tafesh et al. (2011) reported that polyphenolic compounds separated from olive mill wastewater have good antibacterial activity against Gram-positive and negative bacteria. The study reported by Thielmann et al. (2017) proved that the extracts of olive leaves and fruits of *Olea europaea* Linné from Mediterranean countries also have higher effective antimicrobial activity in food matrices. In previous studies, the antibacterial effect of OOPE and its action approach against *Cronobacter sakazakii* and *Bacillus cereus* have already been reported by our team (Fei et al., 2018, 2019). Based on this evidence, it is reasonable to assume that OOPE can be used to inhibit *L. monocytogenes* as a potential natural bacteriostatic substance.

The purposes of this study were to evaluate the antibacterial activity of OOPE against *L. monocytogenes* and to elucidate the possible action approach through investigating the changes in intracellular adenosine 5′-triphosphate (ATP) concentration, cell membrane potential, bacterial protein, cell DNA, and cell morphology after treatment with OOPE.

## Materials and Methods

### Olive Oil Polyphenol Extract Materials

OOPE was provided by Shanghai Kai Da Biotechnology Co. Ltd. (Shanghai, China). The chemical compositions of OOPE include moisture (<7%), total polyphenols (≥30%), hydroxytyrosol (≥6%), tyrosol (≥0.8%), phenolic acids (≥1.5%), and ethanol (<0.1%).

### Bacterial Strain and Culture Condition

A total of eight *L. monocytogenes* strains were used in this study; among them, *L. monocytogenes* CMCC 54004 was obtained from the National Center for Medical Culture Collections (CMCC) of China, and the other seven strains were isolated from food samples. All isolates were used to assess the minimum inhibitory concentration (MIC), whereas *L. monocytogenes* CMCC 54004 was used to analyze the action approach of OOPE against *L. monocytogenes*. The strains were stored in Luria-Bertani (LB) broth with 20% glycerol (v/v) at −80°C. After being cultured in LB broth medium at 37°C with shaking at 150 rpm for 24 h, the strains were streaked onto tryptic soy agar (TSA) plates and continued to be incubated at 37°C for 24 h. A typical colony was selected and inoculated into LB broth at 37°C for 24 h to obtain the pure cultures of *L. monocytogenes* (Dias et al., 2018).

### Determination of Minimum Inhibitory Concentrations

The MICs of OOPE against eight *L. monocytogenes* were determined using the agar dilution method according to previous report (Fei et al., 2018). *L. monocytogenes* cells were treated by different concentrations of OOPE (10, 5, 2.5, 1.25, 0.625, 0.3125, 0.156, and 0.078 mg/ml), respectively, and 0.1 mg/ml ampicillin was used as the positive control. Two microliters of tested bacteria cultures was dripped onto the TSA plate, dried, and incubated at 37°C for 24 h. The MIC was considered as the lowest concentration of OOPE, at which the visible growth of *L. monocytogenes* was inhibited completely.

### Reduction of *L. monocytogenes* CMCC 54004 by Olive Oil Polyphenol Extract in Normal Saline and Luria-Bertani

*L. monocytogenes* CMCC 54004 was cultured in LB broth at 37°C for 24 h, and then the density of strains was adjusted to about 10^8^ CFU/ml with sterile normal saline (NS) and diluted to about 10^6^ CFU/ml in LB as working cultures, respectively, according to the method reported by Bharitkar et al. (2014) with minor modifications. OOPE was dissolved in the working cultures (NS and LB) to obtain final concentrations of 0 MIC, 1 MIC, and 2 MIC. Bacteria were further cultured, and bacterial suspensions were collected from NS and LB after 0.5, 1, 3, 5, and 7 h, respectively. The diluted mixtures (0.1 ml) were incubated on TSA at 37°C for 24 h, and the survival population of *L. monocytogenes* CMCC 54004 was calculated.

### Measurement of Intracellular Adenosine 5′-Triphosphate Concentrations

Based on the method described by Chen et al. (2017), the changes in intracellular ATP concentrations of *L. monocytogenes* CMCC 54004 after treatments with OOPE was measured. The final *L. monocytogenes* CMCC 54004 cell concentration was diluted to be 10^8^ CFU/ml with NS. The ATP assay kit (Beyotime Bioengineering Institute, Shanghai, China) was used to determine intracellular ATP concentration, and all processes were operated on an ice box. Two milliliters of cell suspensions containing different concentrations of OOPE (0 MIC, 1/4 MIC, 1/2 MIC, 1 MIC, and 2 MIC) was incubated at 37°C for 30 min and then mixed with lysis solution. The supernatant was obtained after centrifugation at 11,269 × *g* for 5 min and stored in an ice box to prevent the loss of ATP. Both ATP test solution and supernatant were added to a colorless transparent 96-well plate (Corning Institute, USA) to determine intracellular ATP concentration with an Infinite 200 PRO multifunctional microplate reader (Tecan, Grodlg, Austria).

### Measurement of Membrane Potential

The measurement of membrane potential was performed as previously described by Bharitkar et al. (2014). Bis-(1,3-dibutylbarbituric acid) trimethine oxonol [DiBAC_4_(3); Beijing Solarbio Science and Technology Co. Ltd., Beijing, China] was used as a membrane potential-sensitive fluorescent probe. Bacterial suspension and DiBAC_4_(3) fluorescent probe were added in a black and opaque 96-well plate (Corning Institute, USA) to balance at 37°C for 30 min. OOPE was then added in the 96-well plate to adjust the concentration to 0 MIC, 1 MIC, and 2 MIC, respectively. The fluorescence intensity of each well was recorded with a multifunctional microplate reader (Infinite 200 PRO, Tecan, Grodlg, Austria) under the condition of the excitation wavelength of 492 nm and emission wavelength of 515 nm at 37°C. The value of relative fluorescence units (RFUs) was recorded as the result of membrane potential.

### Sodium Dodecyl Sulfate-Polyacrylamide Gel Electrophoresis

The effect of OOPE on the bacterial protein was analyzed using the sodium dodecyl sulfate-polyacrylamide gel electrophoresis (SDS-PAGE) method as described by Chen et al. (2017). Approximately 10^7^ CFU/ml of *L. monocytogenes* CMCC 54004 cells was treated with OOPE at 0 MIC and 1 MIC in NS at 37°C. The bacterial suspensions were withdrawn every 3 h (3, 6, 9, and 12 h), washed three times using NS, and mixed with SDS-PAGE loading buffer (pH 6.8; 1.0 M tris-HCl, 10% glycerol, 2% SDS, 10% β-mercaptoethanol, and 0.1% bromophenol blue). After heating in boiling water bath for 10 min, proteins were separated by SDS-PAGE using a concentrated gel (5%) and a separated gel (15%). Finally, gels were stained with Coomassie Brilliant Blue R250 (Sigma, USA). The image was taken with the HP scanner (HP 1000, USA).

### Transmission Electron Microscopy

The cellular morphology of *L. monocytogenes* CMCC 54004 cells after treatments with 0 MIC, 1 MIC, and 2 MIC of OOPE was observed using the H-7650 transmission electron microscope (TEM) (Hitachi, Tokyo, Japan) as described by Li et al. (2016). After treatments with different MICs of OOPE for 4 h, *L. monocytogenes* CMCC 54004 cells were centrifuged at 5,008 × *g* for 5 min and prefixed with 2.5% glutaraldehyde at 4°C for 2 h. The pellets were rinsed with 0.1 M sodium phosphate buffer (pH 7.2) three times and postfixed with 1% osmium tetraoxide for 120 min and then rinsed three times again. The samples were dehydrated with different concentrations of ethanol (50, 70, 90, and 100%) for 10 min and infiltrated with the mixture of 100% acetone and Epon resin overnight at room temperature. The infiltrated samples were embedded in Epon Lx-112 (Ladd Research, Williston, VT) and polymerized at 70°C for 12 h. Next, the samples were cut into sections of 50–60 nm, stained with uranyl acetate and lead citrate, and then observed under TEM.

### Agarose Gel Electrophoresis for DNA Fragmentation

The effect of OOPE on the *L. monocytogenes* CMCC 54004 DNA was measured using agarose gel electrophoresis (AGE) according to previous report (Liu et al., 2011). Approximately 10^8^ CFU/ml of *L. monocytogenes* cells were treated by 0 MIC, 1 MIC, and 2 MIC of OOPE in NS at 37°C. The bacterial suspensions were treated for 2, 4, and 10 h, respectively. The genomic DNA was extracted using a bacterial genomic DNA extraction kit (Tiangen Biotech Co., Ltd., Beijing, China). The DNA samples were electrophoresed using 1.5% agarose gel at 100 V for 30 min. Finally, the gels were stained with 10 mg/ml of ethidium bromide for 15 min and visualized using a gel imaging system (Bio-Rad, USA).

### Statistical Analysis

Experiments were repeated in triplicate, and data were analyzed using the SPSS 19.0 software (SPSS, Chicago, IL). All data are expressed as the mean values ± standard deviation (SD). The statistical differences at 5% significance level among means were determined by one-way analysis of variance (ANOVA).

## Results

### Minimum Inhibitory Concentrations of Olive Oil Polyphenol Extract Against *L. monocytogenes* Strains

The results showed that OOPE had inhibitory ability against the growth of eight *L. monocytogenes*. Bacterial colony did not grow when the concentrations of OOPE were higher than or equal to 1.25 mg/ml; therefore, the MIC of OOPE against eight *L. monocytogenes* was 1.25 mg/ml.

### Reduction of *L. monocytogenes* CMCC 54004 by Olive Oil Polyphenol Extract in Normal Saline and Luria-Bertani

**Figure 1:**
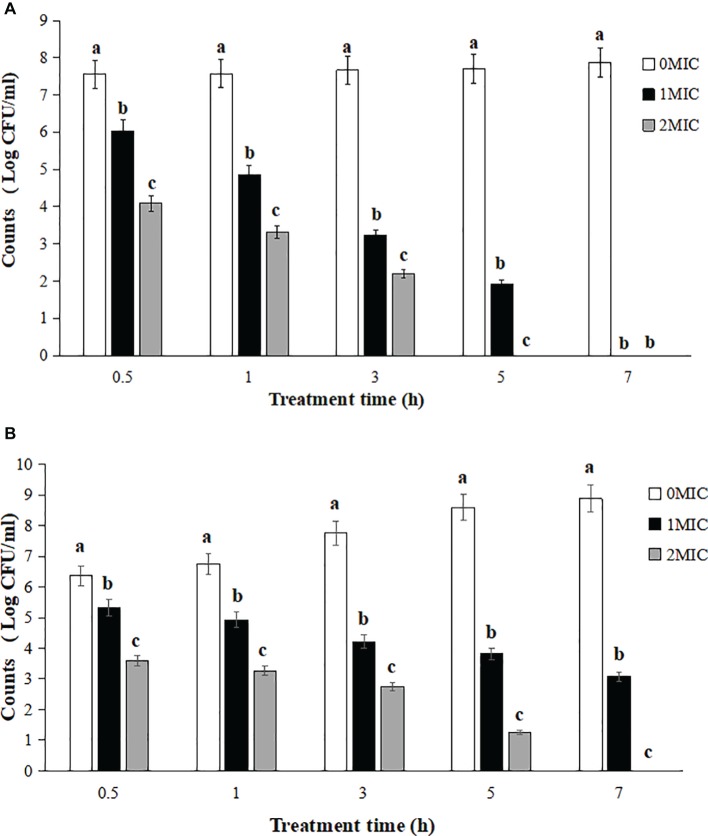
Reduction of *L. monocytogenes* CMCC 54004 with treatments with different concentrations OOPE in NS **(A)** and LB **(B)**. Error bars denote SD. Different letters denote significant differences between treatments within the same incubation time points (*p* < 0.05).

The survival counts of *L. monocytogenes* CMCC 54004 in NS and LB after treatments with 1 MIC and 2 MIC of OOPE are shown in Figure 1. The results showed that the growth of *L. monocytogenes* treated by 1 MIC of OOPE were completely inhibited in NS after 7 h (Figure 1A). Meanwhile, *L. monocytogenes* could be completely inhibited after a treatment with 2 MIC of OOPE in LB after 7 h (Figure 1B).

### Determination of Intracellular Adenosine 5′-Triphosphate Concentrations

**Figure 2:**
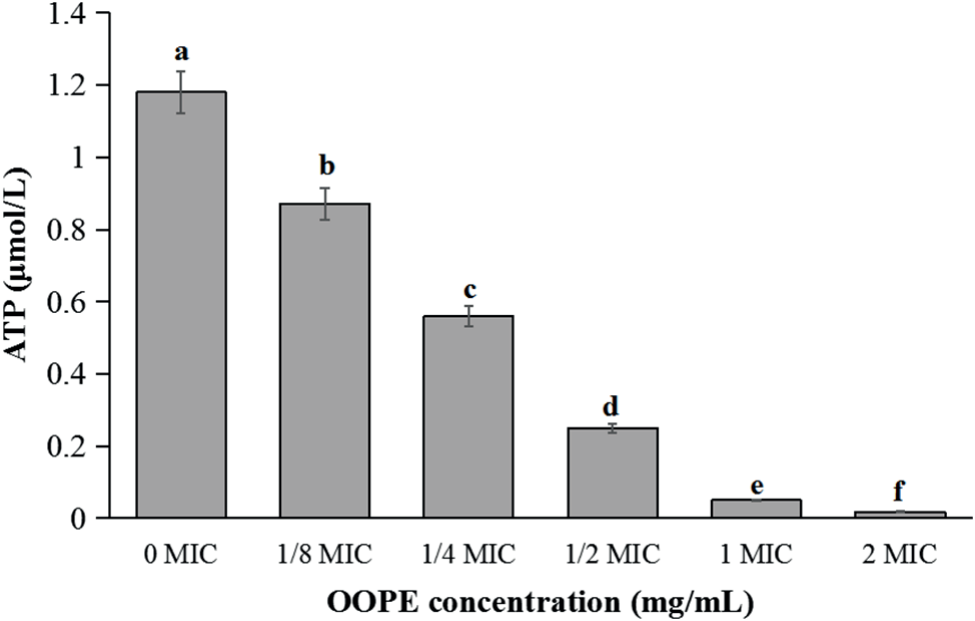
Differences in intracellular ATP concentrations of *L. monocytogenes* CMCC 54004 following treatments with OOPE at 0 MIC, 1/8 MIC, 1/4 MIC, 1/2 MIC, 1 MIC, and 2 MIC. Values represent the means of independent triplicate measurements. Error bars denote SD. Different letters denote significant differences between treatments within the same incubation time points (*p* < 0.05).

As shown in Figure 2, the intracellular ATP concentration of *L. monocytogenes* CMCC 54004 was significantly reduced after treatments with different concentrations of OOPE for 30 min (*p* < 0.05), compared to the control group. Leakage of ATP in the cells of *L. monocytogenes* with the treatment of 2 MIC OOPE was detected to have lower ATP concentration than those of the control and 1 MIC OOPE. In addition, as the concentration of OOPE treatment increases, the intracellular ATP concentration in *L. monocytogenes* cell decreases.

### Changes in Membrane Potential

**Figure 3:**
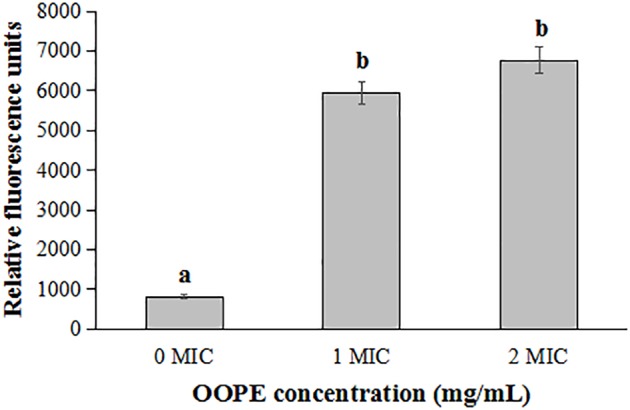
Differences in membrane potentials of *L. monocytogenes* CMCC 54004 following treatments with OOPE at 0 MIC, 1 MIC, and 2 MIC. Values represent the means of independent triplicate measurements. Error bars denote SD. Different letters denote significant differences between treatments within the same incubation time points (*p* < 0.05).

As shown in Figure 3, compared with the control group, the fluorescence intensities of *L. monocytogenes* CMCC 54004 cells treated by 1 MIC and 2 MIC of OOPE were significantly increased (*p* < 0.05). It indicated that OOPE can cause the depolarization of *L. monocytogenes* cells. In addition, there is no difference in fluorescence intensity between the cell treatments with 1 MIC and 2 MIC of OOPE (*p* > 0.05).

### Sodium Dodecyl Sulfate-Polyacrylamide Gel Electrophoresis Analysis of Bacterial Proteins

**Figure 4:**
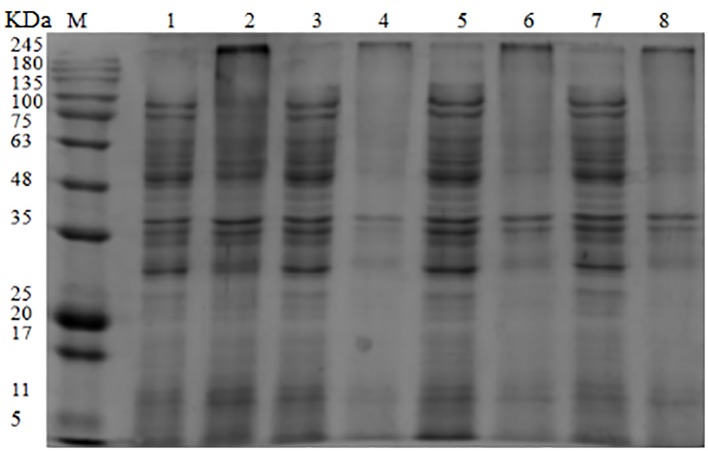
SDS-PAGE analysis of *L. monocytogenes* CMCC 54004 proteins treated with OOPE at 0 MIC and 1 MIC. Lane M: marker. Lanes 1, 3, 5, and 7: treated with 0 MIC of OOPE for 3, 6, 9, and 12 h, respectively. Lanes 2, 4, 6, and 8: treated with 1 MIC of OOPE for 3, 6, 9, and 12 h, respectively.

The SDS-PAGE image illustrated the effect of OOPE treatment on bacterial proteins of *L. monocytogenes* CMCC 54004 (Figure 4). The result showed that compared with the control group, the bacterial protein bands of *L. monocytogenes* CMCC 54004 treated by 1 MIC of OOPE begin to weaken after 3 h. As OOPE treatment time increases, the bacterial protein bands become weaker. In addition, most of the bacterial protein bands of *L. monocytogenes* disappeared after treatment with OOPE for 6 h.

### Transmission Electron Microscope Observation of Cell Morphology

**Figure 5:**
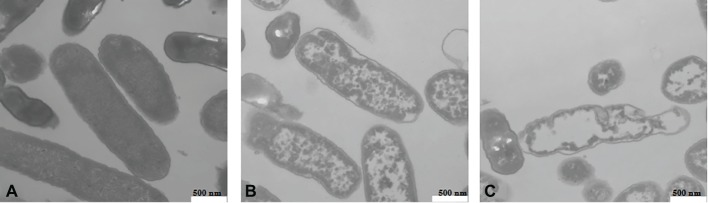
TEM images of *L. monocytogenes* CMCC 54004 cells (40,000×) **(A)** untreated for 4 h, **(B)** treated with 1 MIC of OOPE for 4 h, and **(C)** treated with 2 MIC of OOPE for 4 h.

The cell morphology of *L. monocytogenes* CMCC 54004 cells treated by different levels of OOPE (0 MIC, 1 MIC, and 2 MIC) was observed (shown in Figure 5). The untreated strains showed regular cell morphology, with a normal short rod shape, intact cell structure, and a smooth and compact surface (Figure 5A). After treatment with 1 MIC of OOPE for 4 h, the cells showed morphological damage such as detachment of the cytoplasmic membrane from the cell wall, leakage of intracellular components, and deformation of cell (Figure 5B). Meanwhile, the cells treated by 2 MIC of OOPE displayed more severe cell collapse and leakage (Figure 5C).

### DNA Cleavage Analysis

**Figure 6:**
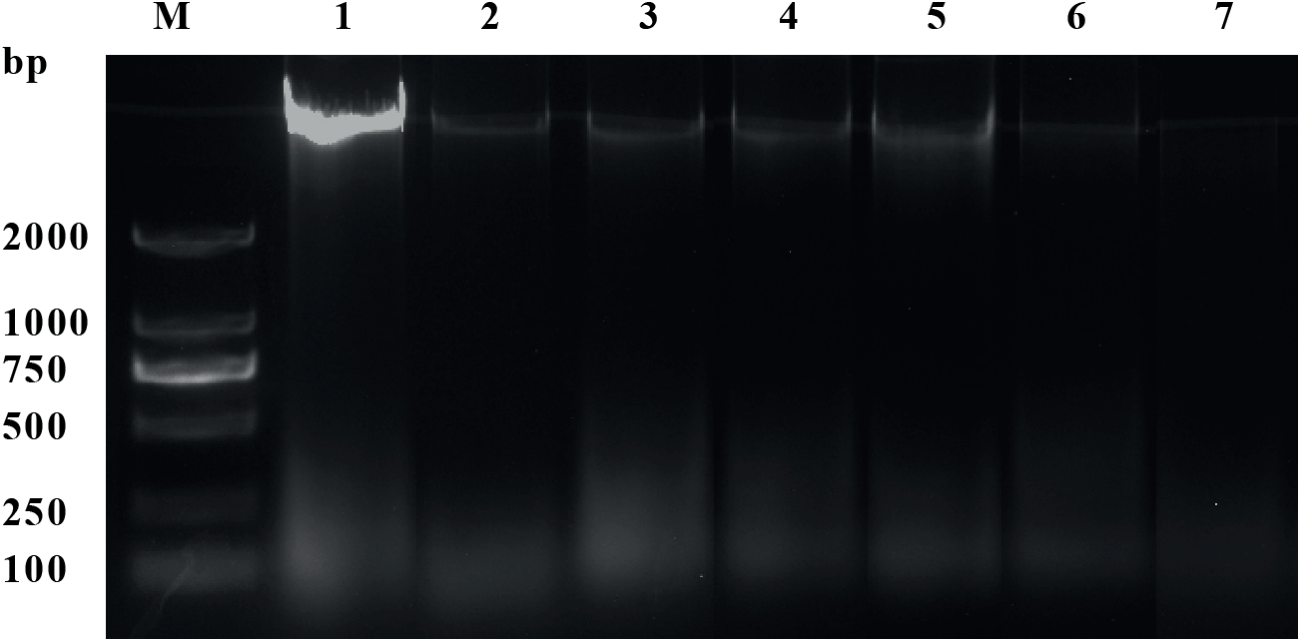
DNA cleavage activity of *L. monocytogenes* CMCC 54004 strains treated with OOPE at 0 MIC, 1 MIC, and 2 MIC. Lane M: marker. Lane 1: control group. Lanes 2–4: treated with 1 MIC of OOPE for 2, 4, and 10 h, respectively. Lanes 5–7: treated with 2 MIC of OOPE for 2, 4, and 10 h, respectively.

As shown in Figure 6, compared with the control group, the DNA bands of *L. monocytogenes* CMCC 54004 after exposure to OOPE were faint and became much fainter as the concentration of OOPE increased. Furthermore, after treatment with 2 MIC of OOPE for 4 h, the DNA bands of *L. monocytogenes* disappeared.

## Discussion

Phenolic products consisting of polyphenol, flavonoids, and tannic acid have been reported to have satisfactory antibacterial activity against food-borne pathogens (Borges et al., 2013). Some studies have evaluated the antibacterial effects of ferulic acids, phenyllactic acid, and sugarcane bagasse extract (mainly containing phenolic substances) against *L. monocytogenes* strains, with MIC of 1.25 mg/ml (Zhao et al., 2015; Ning et al., 2017). In addition, the MICs of gallic acid, sugar beet molasses polyphenols, and cardoon polyphenols against *L. monocytogenes* strains were reported to be 1.60, 5, and 2.5–10 mg/ml, respectively (Borges et al., 2013; Chen et al., 2017; Dias et al., 2018). In this study, the MIC of OOPE against eight *L. monocytogenes* strains was 1.25 mg/ml, which suggested that the bacteriostatic activity of OOPE was worth affirming compared to the above natural extracts.

In this study, the intracellular ATP concentration was significantly reduced after treatments with OOPE, which was consistent with the antibacterial action of OOPE against *C. sakazakii* and *B. cereus* (Fei et al., 2018, 2019). Similarly, Bendali et al. (2008) found that the antibacterial effect of the phenolic compounds of mangosteen against *L. monocytogenes* is related to the decrease in intracellular ATP level and suggested that the loss of ATP was allowed to be released during formation of pore complexes in the cells. In addition, Khan et al. (2017) reported that the loss of inorganic phosphate and K^+^ in cells caused the reduction of intracellular ATP because the cells will consume available ATP in order to reaccumulate these ions. Shi et al. (2016) considered that the increased cell membrane permeability after treatments with natural product resulted in the release of ATP.

Resting membrane potential, as an important predictor of cell survival, was associated with cell antibiotic uptake and bactericidal action (Bot and Prodan, 2009). Our results showed that OOPE caused the immediate depolarization of *L. monocytogenes* cell membranes, which was considered as one of the important bacteriostatic pathways. Similarly, cell membrane depolarization was found in *C. sakazakii* and *Escherichia coli* cells after exposure to lipoic acid and carvacrol and was considered to be associated with less negative charges inside the cells (Silverman et al., 2003; Shi et al., 2016; Khan et al., 2017).

Differences in bacterial protein contents between untreated cells and ones treated with natural-occurring substance can be used to reveal the possible antibacterial action (Wang et al., 2015). Current studies suggested that after treatments with OOPE, the protein levels of *L. monocytogenes* cell were significantly reduced or almost disappeared. A similar action approach has been found by Chen et al. (2017), who illuminated that the bacterial protein bands of food-borne pathogens, including *Staphylococcus aureus*, *L. monocytogenes*, *E. coli*, and *Salmonella typhimurium*, became slighter and even disappeared after treatments with beet molasses polyphenols. Furthermore, the decrease in bacterial protein was considered to be related to reduction in protein synthesis and protein loss due to the increase in membrane permeability (Zeng et al., 2010; Fei et al., 2018).

TEM observation could help visually reveal the changes in cell morphology and cytoplasm of tested cells (Xing et al., 2009). After treatments with OOPE, the cell morphology of *L. monocytogenes* was severely destroyed and was accompanied by a large leakage of cell fluid, leading to cell death. Similar results have been reported by Barbosa et al. (2015), who found cell lysis, damage of cell wall, and leakage of cell contents occurred in *L. monocytogenes* treated by oregano essential oil containing thymol and carvacrol. In addition, the study of Borges et al. (2013) indicated one of the important reasons why ferulic and gallic acids can inhibit the growth of food-borne pathogenic bacteria was the irreversible changes in membrane change and leakage of intracellular components.

DNA, as the main genetic material, is considered the cornerstone of life’s activities and is closely related to the bacterial growth, development, and inheritance (Cui et al., 2018). Combining the results of DNA fragmentation in this study, it can be supposed that OOPE may inhibit DNA synthesis or promote the cleavage of DNA of *L. monocytogenes*. Similarly, clove oil-containing phenols can inhibit the growth of *L. monocytogenes*, and DNA fragmentation appeared in treated cells (Cui et al., 2018). Besides, Wang et al. (2017) suggested that the phenolic components not only reduced the amount of DNA in cells by increasing cell membrane permeability and destroying the cell morphology but also bound to the minor groove of genomic DNA, resulting in changes in the secondary structure and morphology of DNA.

In conclusion, the present study indicated that OOPE has significant antibacterial activity against *L. monocytogenes*, and its antibacterial action was related to lower intracellular ATP, cell depolarization, decrease in bacterial protein and DNA, and cell fluid leakage due to destruction of cell morphology. These findings demonstrated that OOPE has potential as a food preservative to reduce the risk of contamination of *L. monocytogenes*. However, OOPE safety and specific application in food preservation need to be researched in the future.

## Data Availability

All datasets generated for this study are included in the manuscript.

## Author Contributions

PF, SG, LG, WJ, and WX conceived and designed the experiments. QS, SG, and XB performed the experiments. LG, PF, SG, and QS generated and analyzed the data. LG, PF, and QS wrote the paper.

### Conflict of Interest Statement

The authors declare that the research was conducted in the absence of any commercial or financial relationships that could be construed as a potential conflict of interest.
